# Consumer Attitudes Towards Environmental Concerns of Meat Consumption: A Systematic Review

**DOI:** 10.3390/ijerph16071220

**Published:** 2019-04-05

**Authors:** Ruben Sanchez-Sabate, Joan Sabaté

**Affiliations:** 1 Centro de Excelencia en Psicología Económica y del Consumo (CEPEC), Núcleo Científico y Tecnológico en Ciencias Sociales y Humanidades, Universidad de La Frontera, Temuco 4811230, Chile; 2 School of Public Health. Loma Linda University, Loma Linda, CA 92350, USA; jsabate@llu.edu

**Keywords:** consumer attitudes, meat avoiders, meat reducers, environmental concerns, global warming, climate change, sustainability, ecology, planetary health

## Abstract

Meat consumption is a major contributor to global warming. Given the worldwide growing demand of meat, and the severe impact of meat production on the planet, reducing animal protein consumption is a matter of food security and public health. Changing consumer food behavior is a challenge. Taste preferences, culinary traditions and social norms factor into food choices. Since behavioral change cannot occur without the subject’s positive attitude based on reasons and motivations, a total of 34 papers on consumer attitudes and behavior towards meat consumption in relation to environmental concerns were examined. The results show that consumers aware of the meat impact on the planet, willing to stop or significantly reduce meat consumption for environmental reasons, and who have already changed their meat intake for ecological concerns are a small minority. However, environmental motives are already appealing significant proportions of Westerners to adopt certain meat curtailment strategies. Those who limit meat intake for environmental reasons are typically female, young, simply meat-reducer (not vegan/vegetarian), ecology-oriented, and would more likely live in Europe and Asia than in the U.S.

## 1. Introduction

Worldwide demand for meat and other animal products is increasing due to rising incomes, growing populations and other sociocultural factors [1,2]. This trend is a global problem because meat production is a major responsible for global warming and environmental degradation [1,3,4,5,6]. The livestock industry pollutes freshwater with antibiotics, hormones and chemical substances among others, depletes freshwater availability, contributes to the loss of biodiversity, and is a major source of anthropogenic greenhouse gas emissions [1]. Consequently, finding ways to make diets more sustainable by reducing animal protein consumption has become a matter of food security and thus, a public health issue [7].

Changing consumer food behaviors is a challenge. They are the result of strongly held factors like taste preferences, culinary traditions and social norms [8]. Health behavior theorists have described the stages a person undergoes when trying to adopt healthy behaviors. They cite that behavioral change can only occur with the adoption of a positive attitude based on reasons and motivations [9]. It is therefore relevant to know if environmental reasons can prompt individuals to reduce or avoid meat consumption.

In Western societies, meat-based diets are the norm. Meat avoiders like vegans and vegetarians represent a small minority. For instance, in the United States and the United Kingdom, vegetarians account for significantly less than 5% of the population [10]. The motivations of converted vegans and vegetarians—those raised on a meat-based diet—have been described as non-static and related to health, economy, environment, society and culture, ethics and religion [11]. Vegetarians can be categorized in two large groups: health oriented and ethically motivated [12]. This is because the most prevalent motivations among vegetarians are health and animal welfare [13,14,15,16,17]. Environmental reasons, on the contrary, are important to a small fraction of vegetarians [11].

Another group of consumers to consider are those not ready to give up meat, but who have, or are willing to consider reducing meat consumption. These are known as meat-reducers or flexitarians. Contrary to vegans and vegetarians who have been studied for decades, meat-reducers have received scant attention [18].

The goal of this systematic review is to enhance our understanding of consumer attitudes on meat consumption in relation to environmental sustainability in order to support potential public health interventions oriented towards meat intake reduction. We looked into the three main stages of behavioral change process as proposed by Glanz et al.’s [9]: awareness (precontemplation), willingness (contemplation and preparation) and change (action, maintenance and termination). Having a general overview of the three stages should give public health professionals a general understanding of the role environmental reasons may play in the food eating behavior change process. Thus, this systematic review aims to answer the three following research questions: (1) Are people aware of the environmental impact of meat production and consumption? (2) Are people willing to stop or reduce meat consumption based on environmental concerns? and (3) Have ecological/environmental concerns been the motivation for people who have altered their meat consumption?

## 2. Materials and Methods

This systematic review was reported following the PRISMA (Preferred Reporting Items for Systematic Reviews and Meta-Analyses) guidelines [19] We performed a preliminary search in Google Scholar for articles that reported data on at least one of the following three topics: people’s awareness of the environmental impact of meat production and consumption; people’s willingness to stop or reduce meat consumption because of environmental concerns; and people who have already stopped or reduced meat consumption because of environmental reasons or motivations (diet change).

This initial search allowed us to identify a series of keywords that we later used to conduct a literature search of the Web of Science (WOS) Core Collection in March 2018. A separate query was conducted for each topic (awareness, willingness and diet change). Each query consisted of a series of search strings that combined no more than three terms each from one of the following categories: consumer related, meat related, and planet related. For example, one query looked like this: consumer attitudes AND meat AND climate change.

Thus, for “awareness” we used a series of search strings that combined the following terms: “consumer/people attitudes/perceptions” AND “meat”/“livestock” AND “climate change”/“GHG emissions”/“global near/2 warming”/“environment”/“water near/3 use”/“land near/3 use”. Similar search strings were used for “willingness” and “change”.

**Figure 1:**
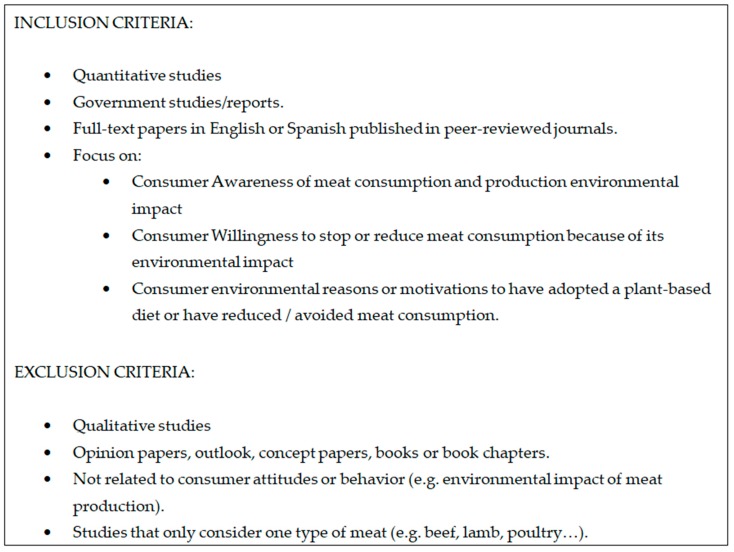
Eligibility criteria.

The screening process was completed by both authors independently to reduce bias. It comprised three stages for each one of the three topics considered. First, articles and abstracts were screened. Citations that met the eligibility criteria (Figure 1) were imported to the reference manager Zotero. Second, selected citations were read in full to make a final decision on their relevance for any of the three topics considered, and to locate new relevant articles that had not been found by the WOS search. Third, these first two steps were conducted for the new bibliography until no new eligible references were detected. The few articles considered pertinent by only one reviewer were included or discarded after a discussion between the two coauthors. The search for “awareness” yielded a total of 14 articles that met the eligibility criteria. The search for “willingness” yielded a total of 16 articles that met the eligibility criteria. And the search for “diet change” yielded a total of 17 articles that met the eligibility criteria. This systematic review rendered a total of 34 articles since some publications were relevant for more than one topic. Pertinent data from these articles was abstracted in tables with categories including: study design, sample characteristics, question or dependent variable and covariates effects, among other relevant information.

## 3. Results

### 3.1. People Awareness of the Environmental Impact of Meat Production and Consumption

The results from 14 articles that examined awareness of the negative impact meat production and consumption have on the environment are summarized in Table A1, presented in the Appendix A at the end of this dcocument. In short, the main findings are: (1) aware consumers are a minority; (2) consumers either underestimate or ignore the potential of either stopping or reducing meat production and consumption to reduce the anthropogenic impact on the environment; and (3) it is not clear for the consumer that a vegetarian diet is more environmental friendly than a diet including meat.

Consumer awareness of the meat environmental toll has been studied in Belgium, Finland, Germany, the Netherlands, Portugal and the United States using different methods. The percentages of aware participants ranged from 23% to 35% across studies [20,21,22]. One study in which subjects received prior information, the percentage jumped to 58% [23]. Another study required respondents to list concrete impacts of meat production on the planet: only 24% named “pollution” and 20% “erosion of natural resources” [24]. Another study showed a tendency toward a neutral opinion on the negative environmental impact of meat [25]. And regarding behaviors that damage the earth, one study showed that consumers rarely (less than 10%) thought of “meat eating” [26].

Consumer estimation of meat production and consumption toll on the environment was studied in Australia, Belgium, the Netherlands, Switzerland, the United Kingdom, and the U.S. Only two studies specifically queried participants on meat production. Less than half (38%) agreed that changing animal husbandry can counter climate change [20], but still its toll was underestimated relative to other activities like transport, even when prior information on meat and the environment was given [23]. All other studies focused on meat consumption reduction. Percentages of participants agreeing with it as a way to help the environment varied between 18% to 29% across studies [27,28,29]. Percentages of subjects that considered it an effective way to alleviate climate change varied from 5% to 64%. This big range can be explained by different methodological and geographical factors across studies. Still, reducing meat consumption was usually considered the least or second least effective when compared to other options [26,30,31]. Still consistent with this finding, the only longitudinal study found by the reviewers showed that participants gave slightly higher effectiveness to meat reduction in the follow-up survey four years later [32]. Finally, it is not clear to consumers that a vegetarian diet is more environmentally friendly than a diet with meat [25].

Not all studies report on covariate effects. From those which do, the gender variable is the most frequent one. Women are more conscious about the negative impact meat has on the environment [22,25], and thus, they perceive a higher effectiveness in reducing meat consumption to alleviate climate change than men [26,30,31,32,33]. One study found that the only important covariates were the frequency of meat intake and already established concerns about the environment. As meat intake went up, the perceived effectiveness of meat reduction went down. But the subjects who held a strong belief in human causation of climate change assigned a positive association between eating less meat and helping the planet. Other covariates like age and level of education presented no correlations [30]. Another study also showed no correlations of awareness with age, but surprisingly, neither with gender nor with meat consumption frequency [28].

### 3.2. People Willingness to Stop or Reduce Meat Consumption Because of Environmental Reasons or Motivations

The results from 15 papers plus a European Union Report (EUR) that examined people willingness to stop or reduce meat consumption for environmental reasons are summarized in Table A2 (see Appendix B). The main findings are: (1) those motivated by ecological concerns to reduce meat intake are a minority, and (2) meat curtailment is among the least preferred personal options to counter climate change.

When no prior information on the meat environmental toll was given, participants from Finland, Germany, The Netherlands, Switzerland and the U.S. willing to stop or reduce meat consumption because of environmental reasons ranged from 12.8% to 25.5% [22,25,33]. Reducing meat intake was usually the least chosen option to curb climate change [26,30]. Belief in the negative impact of meat on the planet associated positively with willingness to change meat consumption in three studies [26,30,33]. One study also revealed a positive association between consciousness, understood as cognitive and affective awareness of the environmental toll of meat, with willingness to reduce meat consumption [22]. Another study that specifically distinguished between belief and actual knowledge on the effectiveness of meat reduction for climate change mitigation, showed that while belief was positively associated with willingness, knowledge was not [26]. Only one study explicitly reported that education and age were not related to willingness [33].

Eight studies conducted throughout Belgium, Germany, The Netherlands, Portugal, Sweden and the U.S., and the EUR did provide information to the participants connecting meat production and consumption with the environment before the data collection. The results show disparate percentages of people willing or maybe willing to reduce meat consumption for environmental reasons. If simply asked for their willingness to make such a dietary change, participants “certainly willing” were a small minority (5–18%), while those “maybe willing” were 41% [20,25]. Regarding agreement with certain direct meat curtailment strategies, percentages varied widely (15–60%) depending on the strategy considered. Meat substitution for vegetables was significantly less popular than meat reduction, but the latter was still among the least preferred options unless compared with eating insects or meat substitutes [21,23,24]. In one study, participants did not find altering meat consumption easy to do [34].

The EUR [35] reported that about 50% of Europeans would be willing to replace most of the meat they eat with vegetables, and 80% of them would be willing to eat less meat but of certified origin. Considering some countries separately, the UK, the Netherlands, Denmark, Finland and Belgium present lower percentages of people willing to replace meat with vegetables (29–49%) and of people willing to consume less meat but of certified origin (62–73%) than countries like Portugal, Spain, Italy and Romania in which percentages range from 53% to 69% and 83% to 89% respectively.

Covariate effects are similar to those presented in the awareness section. Being female is usually a strong predictor of willingness to decrease meat consumption or choose meat-free menus [21,29,31,35,36]. Meat consumption frequency and positive attitudes to meat are negatively associated with willingness to eat it less [21,24,31,37]. Ethnicity and culture can strongly influence willingness. Turks living in the Netherlands were less willing to alter meat consumption than Chinese and Native Dutch [36]. Mediterranean Europeans responded more positively to replacing most of the meat with vegetables (56% average) and to reduce meat consumption (86%) than Northern Europeans (46% and 80%, respectively) [38]. Regarding income, one study presented a negative association between affluence and willingness [38]. Age and education, on the contrary, had in general no influence [31].

Finally, the effect of information on meat and the environment on willingness is less clear. In one study, it could be seen that prior information increased the percentage of people willing to eat less meat from 12% to 18% [25]. In two other studies, information did not alter the number of participants willing to choose meals with less or no meat [21,29]. However, one study reported that participants concerned for the environment and/or already aware before the experiment about the negative impact of meat, were more likely to support meat curtailment strategies [21]. Still, another study found that pro-environmental beliefs had no significant predictive value [29]. In any case, it should be kept in mind that each study provided participants with different types, degrees, and formats of information on the meat environmental toll and thus, generalizing results is not recommendable.

### 3.3. Meat Consumption Changes for Environmental Reasons

The results from 17 articles that examined motivations for limiting meat consumption are summarized in Table A3 (Appendix C). The main findings show that those who have already adopted a meatless diet or have already reduced its consumption are: (1) a small minority among samples from the general population, and a significantly bigger one among certain population groups; and (2) female, most likely young, partial meat limiters and reside in Europe.

The studies reviewed referred to people who follow a low or no-animal product diet in two different ways: (1) vegans and vegetarians; and (2) “meat avoiders”, “animal product limiters”, and similar expressions. This fact directly affected the wording of questions and sentences that participants had to answer or rate. Thus, some studies looked for reasons for being “vegan”, “vegetarian” or something similar like “semi-vegetarian”, while other studies searched for reasons for “avoiding meat”, “reducing meat consumption” or any other wording that means the curtailment of animal products consumption. It is necessary to bring attention to this point because veganism and vegetarianism are not only a diet choice but an identity [39]. Deciding to become a vegetarian is a much more complex process than simply opting for reducing or avoiding meat consumption, or even adopting a plant-based diet.

Studies that specifically asked for reasons or motives for being vegan/vegetarian were all conducted in the U.S. Those who indicated environmental concerns were few (>3.2%) [39,40] in recent surveys with a general population of vegans/vegetarians. However, among specific population groups environmental vegan/vegetarians were significant minorities: 14% in the case of marathon runners [41], and 32.1% in the case of women physicians surveyed two decades ago [42]. Other research conducted in the U.S. and Finland showed that vegans, vegetarians and semi-vegetarians tend to agree with and give a moderate importance to the protective benefits of a vegetarian diet towards the environment [43,44].

Only a few consumers (4–19%) indicated environmental concerns for having reduced or avoided meat intake in studies conducted in Belgium, The Netherlands and the U.S. [23,45,46]. However, when specific population groups and certain meat curtailment strategies are considered the percentage of environmental meat reducers or avoiders increases. More than a 50% of a general population sample from The Netherlands reported to have “one meat-free day a week” and “smaller meat portions” at least once a month [47]. Other studies showed that meat avoiders/reducers gave a moderate importance to environmental concerns in their meat purchasing and consumption habits [48]. Those who considered ecology important were the 38.2% of a Dutch sample [45,49,50]. And 38.1% of university students from eleven Eurasian countries pointed to the environment as their major reason for meat avoidance [51].

Reported covariate effects across studies, and research on specific groups like vegans, portray those who limit meat consumption because of the environment as female, young, semi-vegetarian/meat reducer, ecology-oriented, and more likely living in Europe and Asia than in the U.S. Four studies that specifically asked participants to indicate their main reason for meat reduction or avoidance further reflect this profile [39,40,49,51]. Once more, women proved more likely to reduce meat intake because of the environment than men. This was true for Euromerican women [48,49], and for a multiethnic sample from The Netherlands [47,52]. Studies rarely reported age as a significant covariate. However, considering the one study that did [43], and the fact that this review found the highest percentage of meat avoiders because of the environment, in a survey of 3433 students attending different universities based in eleven Eurasian countries [51], it appears that young people may be the most motivated by ecology for already having reduced or stopped meat intake. The degree of involvement with food and sustainability, regardless of age, is another covariate that also correlated positively with environmental reasons for meat curtailment [46,47]. Ethnicity, as well, had a significant impact in one study conducted in The Netherlands [36].

Considering only studies published after 2010, vegans and meat limiters may be more likely to be influenced by environmental reasons than vegetarians. Samples from the U.S., Europe and Asia presented much lower percentages (9–21%) of vegetarians that consider sustainability an important factor that shapes their diet than semi-vegetarians (30–49%), light semi-vegetarians (34–44%) or meat limiters in general (41%) [48,49,51,52]. Two studies carried out in the U.S. before the year 2000, add to this pattern: 60.7% of all types of meat limiters including vegetarians [45] and 32.1% of self-described vegetarians indicated ecological concerns as current reason for their dietary choices [42]. An older study in the UK also showed that vegans and meat reducers are more likely to be influenced by environmental reasons than vegetarians [53]. Opposite results to this pattern, meaning that vegetarians reported to be more influenced by ecological concerns than vegans and meat reducers, appeared to a certain extent, in a study conducted in Finland [44]. In any case, more evidence is needed in order to draw conclusions on differences between vegans, vegetarians and meat reducers. Finally, two recent surveys of vegans living in the U.S. yielded very low percentages (2–3.2%) of consumers motivated by the environment [39,40], adding country location as another significant variable to consider.

## 4. Discussion

The reduction of meat production and consumption would alleviate the anthropogenic impact on the environment [1]. Individual choices for diets low in meat and high in vegetables are urgently needed according to the latest scientific evidence [7]. Previous studies have identified two main motivations that prompt people in the West to become vegan or vegetarian: animal welfare and health [14,16,45,54]. Ecological concerns, however, are only relevant to a minority of them [11]. In addition to vegans and vegetarians, there are a significant number of consumers who limit meat consumption. Known as meat-reducers or flexitarians, few studies have explored their motivations for reducing meat intake [18].

Review of the main findings shows that, in the so-called developed countries, those aware of the meat impact on the planet, and those willing to alter their meat consumption for environmental reasons, are a small minority. This result is in line with a previous review of awareness and willingness only [55]. Regarding change, the present review shows that people who altered their meat consumption patterns because of the environment represent also a small minority of the studied samples. Within this minority of people aware, willing, or who have already changed, women are a clear majority. Considering in addition that the reduction of meat consumption tends to be among the least preferred strategies to alleviate climate change when compared to other non-food activities like driving less, it looks like environmental reasons are not a major motive for reducing meat intake for the general Western population.

Giving information on the environmental toll of meat production could be a promising strategy to increase awareness and willingness. Studies that provided participants with such information before the test showed significantly higher percentages of people aware and willing. However, there are two other factors that could very well explain such increases. First, social desirability, i.e., survey respondents’ tendency to give answers they believe will be viewed favorably by researchers or other participants. The second factor, which applies only to the studies reviewed on willingness, has to do with their different designs. Percentages of people willing to alter meat consumption when prior information is given vary from 5% to 80% in the papers reviewed. Such significant disparity could be explained by studies variations in: (1) methodology; (2) the assessed behavioral action state: some studies measured “belief” while others “intention” or “willingness”; (3) the definition of target behavior (it is not the same to aim for a plant-based diet than for eating meat-free meals regularly) and (4) the time frame to adopt the favorable behavior: for instance, having a meat-free meal x times per month or per week. Therefore, it remains unclear how beneficial the strategy of informing the consumer on the meat environmental toll will actually be for the reduction of its intake.

It is also necessary to pay attention to how the information on the meat impact on the environment is usually introduced. The papers reviewed present the environmental problem in a very rational and detached way. By this we mean that prior information given or questions addressed to participants are based on the common-sense supposition that the environment is separate from, and around, humans. As Lakoff [56] has argued, this is a false supposition because humans are an inseparable part of nature. Yet, this mode of thinking and understanding (“frame” in communication sciences parlance) is common in mass media and public policy communications [56], as well as how scientists word the questions they use and how study participants interpret them. Thus, it is necessary to explore how subjects would react to meat curtailment strategies when ecological concerns are presented to them in an emotional fashion. Research on this regard is promising as environmental messages that appealed to emotions and/or values reduced the intentions of participants to eat meat and affected their attitudes towards meat consumption [34,57,58]. However, research on the effects of emotional messages on people’s attitudes and behaviors towards climate change in general has shown that fear-based appeals can backfire and lead to a decrease in participants’ willingness to reduce their carbon footprints [59]. In addition, a longitudinal study conducted in the UK showed that levels of concern and motivation to behaviorally address climate change decrease as time passes from participants’ exposure to climate change communications [60]. Therefore, more research on communication strategies to increase awareness and willingness to alter meat consumption among Westerners is needed.

Surprisingly, despite increased media attention in recent years to the environmental concerns linked to meat consumption, percentages of vegan, vegetarian and meat reducer participants who claim to follow such dietary patterns on environmental concerns have remained largely unchanged in studies conducted after 2010 compared to the few published before 2002 included in this review. This could be explained by the fact that scientific knowledge and even dietary recommendations for reducing meat consumption based on environmental reasons precede the time span (1987–2016) of the studies included in this systematic review [61]. Such knowledge evidently permeated to vegans and vegetarians long before the more recent mass media attention, probably because they have belief systems and/or sources of information outside the mainstream.

The studies reviewed have limitations that should be addressed in future research. The geographical limitation (the fact that the majority of studies were conducted in only a small number of countries of northern Europe and North America) is the most noticeable. The large survey carried out by the European Commission showed big differences in willingness between northern and southern European countries [35,38]. This gives reason to believe that research on awareness, willingness, and change regarding meat consumption in relation to planetary health can yield significantly different results when Mediterranean, Latin American, and the so-called developing countries are considered. Were this the case, such differences could be explained due to cultural and economic determinants.

There are also methodological limitations worth considering when designing future studies. The majority of the studies reviewed used convenience samples. Random samples are better in order to generalize results to the general populations. Another limitation is that we have found only one longitudinal study. Longitudinal studies could be of interest to identify the evolution of the influence environmental reasons may have on subjects throughout their lives. Cultural aspects may not have been sufficiently taken into account. One study noted large differences in willingness and diet change across ethnicities living in the same country [36]. Further research exploring willingness and change could benefit from an understanding of the cultural significance meat has in the culture/society to be studied. For this and the geographical limitation mentioned before, we consider the results of this systematic review hard to generalize cross-nationally.

Future research could incorporate covariates such as gastronomic and hedonistic dimensions of meat intake and people’s cooking skills when examining willingness and change. Previous studies have already shown that people rarely want to give up meat for the pleasure it gives them [27,62,63]. Thus, it is probable that those who do not have the skills to cook palatable meat-free meals, may not reduce its consumption not because they do not want to, but because they do not know how to have an enjoyable food experience without meat. Another covariate to consider in future research is the participant’s social networks. Since eating is a socially regulated behavior [64], such an important dietary change as altering meat consumption may be favored or impeded by, for instance, family and/or significant communities such as churches, vegetarian associations.

## 5. Conclusions

This systematic review reveals a lack of disposition by the general population in Western countries to stop eating meat on environmental reasons. Even for vegans/vegetarians, ecological concerns are more of another motive to further justify their dietary pattern than an original motivation to give up animal products altogether. However, the reviewed evidence also shows that environmental motives are already appealing to significant proportions of Western meat-eaters to adopt certain meat curtailment strategies like meat-free days. This appeal is more prevalent among women and people from certain cultures. Given that dietary habits are not static, and the fact that mass media attention to sustainable food systems and diets is increasing, it is feasible that ecological concerns become a trigger to at least minor reductions in meat consumption for a majority of the Western population, especially for those not motivated by health or animal welfare. Since a small reduction in meat intake among a large proportion of Westerners could mean a significant contribution to reducing the anthropogenic impact on the environment, mass media outlets, public health educators, nutritionists, policy makers, and the food industry may also consider environmental reasons to promote healthy and sustainable diets.

## Appendix A

**Table A1:** People’s awareness of the environmental impact of meat production and consumption.

Title					Outcome Measure: Perceived Environmental Impact		
Author(s), Year	Design; Year Data Collected	Country; Sample	Main Research Question	Provided Information Prior to the Experiment	Question or Dependent Variable	Response or Finding	Effect of Covariates
Campbell- Arvai, 2015 [29] *	Survey in dining halls; unspecified	U.S.; undergraduate students, convenience sample, *N* = 320, 46% men	Food-related environmental beliefs and behaviors	No info	(1) Eating less meat can help the environment. (2) Adopting a vegetarian diet can help the environment	(1) 29% agree; 20% unsure; 51% disagree (2) 22% agree; 13% unsure; 65% disagree Lowest level of agreement compared with other behaviors (e.g., using less packaging, grown locally)	n.a.
Clonan et al., 2015 [28] *	Postal survey; 2009	UK (Nottinghamshire); random sample from electoral registers, *N* = 842, 41% men	Meat consumption attitudes and sustainable meat purchase	No info	To help reduce the impact of climate change, it is better to eat less animal foods (meat, dairy products and eggs).	18% agree 46% unsure 36% disagree	Red and processed meat intake frequency, sustainable meat purchase frequency, gender, age, SES were not significant
Cordts et al., 2014 [25] *	Online experiment; 2013	Germany; quota sample, *N* = 590, 52% men	Consumer response to negative information on meat consumption	Variables measured before info provision (experimental manipulation)	(1) Farming animals and producing animal products (e.g., milk or meat) has a considerable negative environmental impact. (2) A vegetarian diet is more environmentally friendly than a diet including meat.	(1) M = 3.07, SD = 1.12 (1 = do not agree at all to 5 = fully agree) (2) M = 3.10, SD = 1.21 (1 = do not agree at all to 5 = fully agree)	(1) Women agreed more than men (M = 3.19; SD = 1.11; M = 2.95; SD = 1.12; *p* ≤ 0.01) (2) Women agreed more than men (M = 3.23; SD = 1.19, M = 2.98; SD = 1.21; *p* ≤ 0.05)
De Boer et al., 2016 [30]	Nation-wide consumer surveys; 2014	Netherlands and the USA; representative sample *N* = 527 (The Netherlands). Weighted variables: gender, age, level of education, region, and a value-related test score on “mentality-environment”. (efficiency of the weighting 89%, effective sample size 478) *N* = 556 (USA). Weighted variables: gender, age, and level of education (efficiency of the weighting 90%, effective sample size 500) Total = 1083	Consumer awareness of meat consumption environmental impact and their willingness to reduce meat consumption, among other research questions.	No prior info given.	“For each of the following lifestyle- changes, please let us know whether you think this is an effective way of combatting climate change”. The options, which were presented in randomized order, were: “Eat less meat”, “Buy local, seasonal, unprocessed foods (e.g., by going to farmer’s markets)”, “Buy (more) organic foods”, “Drive less”, “Save energy at home (e.g., turning thermostat down, using saving bulbs, air-drying laundry)”, and “Install solar panels on my house”.	Dutch: “eating less meat” option, second less effective 12% recognized the outstanding effectiveness of the less meat option in the eyes of climate experts 46% attributed effectiveness to the “eating less meat” option Americans: “eating less meat” option, the least effective 6% recognized the outstanding effectiveness of the less meat option in the eyes of climate experts 30% attributed effectiveness to the “eating less meat” option	Regular meat eaters assigned lower effectiveness ratings to the less meat and the organic food option, but not to the other options. Belief in human causation and personal importance were associated with assigning higher effectiveness ratings to all the options. The pattern of profile results remained unchanged when gender, age, and level of education were entered as covariates. The analysis revealed that these variables had small effects on the effectivity ratings. Females gave slightly higher ratings than males, especially to the food-related options
Study 1 de Boer, Schösler, et al., 2013 [20] *	Online survey; 2010	The Netherlands; quota sample, *N* = 1083, 50% men	Motivational explanations for responses to the meat-free meal idea	No info before questions	(1) Agriculture and animal husbandry together are one of the major causes of climate change. (2) If agriculture and animal husbandry change the way they work, they can counter climate change.	(1) 23% agree 36% unsure 41% disagree (2) 38% agree 37% unsure 25% disagree	n.a.
Study 2 de Boer et al., 2014 [31] *	Online survey; 2010	The Netherlands; quota sample, *N* = 1083, 50% men	Consumer strategies to reduce meat consumption and its’ association with their willingness to eat meatless meals	As an individual, you can make a big difference to nature and climate protection by choosing one (or more) meals without meat every week.	Did you know that?	64% yes, 36% no	More ‘yes’ responses for older and better educated people
De Groeve, et al., 2017 [21]	Online survey. Two samples. Data collected in 2015 (sample 1) and 2016 (sample 2)	Belgium; Ghent University Business Administration Students; *N* = 429	Assess students support for six less meat initiatives (LMIs) to be implemented in student restaurants.	No prior info given.	Students’ knowledge about the negative impact of meat on the environment	4.66% reported “Very much” 24.4% rather much 36.6% not little, not much 24.4% Little 9.79% Very little	n.a.
Graca, Oliveira, et al., 2015 [24] *	Online survey; 2013	Portugal; convenience sample, *N* = 410, 30% men	Multiple correspondence analysis to identify clusters of meat-related associations	Info provided after the question	Participants responded to an open ended question about how meat consumption may impact nature and the environment	24% pollutes nature and the environment; 20% erosion, disruption, depletion of natural resources; 18% references to mass production, artificial methods; 14% impacts only if unregulated or in excess; 11% does not impact nature and the environment;	n.a.
Lea & Worsley, 2008 [27] *	Postal survey; 2004	Australia (Victoria); random sample, *N* = 223, 48% men	Food-related environmental beliefs and behaviors	No info	Consumers eating less meat’ is important to help the environment	22% agree 22% unsure 56% disagree Lowest level of agreement compared with other behaviors (e.g., using less packaging, grown locally)	n.a.
Pohjolainen et al., 2016 [22]	Postal survey; 2010	Finland; representative sample. *N* = 1890	The level of environmental consciousness among Finnish consumers concerning meat production and consumption	No prior info given.	Participants had to agree or disagree with the following three statements: (1) meat production strengthens climate change significantly more than plant production (2) meat production causes eutrophication significantly more than plant production (3) food production causes significant environmental problems	(1) 35.7% agree; 47% neutral; 17.3% disagree (2) 34.8% agree; 45% neutral;20.2% disagree (3) 35.6% agree; 37.7% neutral; 26.7% disagree	Consumers clustered in six groups depending on their awareness of meat-related environmental questions: Those aware (highly conscious and rather conscious), those resistant to the idea (Resistant), those who give neutral answers (highly unsure and rather unsure) and those “careless conscious”. Among the groups highly and rather conscious, the majority is female (66.2% and 55.3%), two thirds aged between 46–75, 40% or more have tertiary education. When occupation is considered, in both groups more than 40% are not in labor force and blue-collar workers are slightly more represented than white-collar (26.1–21.1%/21.9–19.6%).
Tobler et al., 2011 [33] follow-up study by Siegrist et al., 2015 [32] *	Postal survey; longitudinal study: 2010, follow-up 2014	Switzerland (German- and French-speaking regions); random panel sample, N2010 = 6189, N2014 = 2781, 48% men	Consumer willingness to adopt ecological food consumption	No info	Perceived environmental benefit of eating less meat (maximum of once or twice per week), (1 = very small to 6 = very large)	M = 3.75, SD = 1.71, reducing meat consumption was perceived as having the lowest environmental effect compared with other behaviors (e.g., avoiding excessive packaging or organic food). Longitudinal study; Increase across time (M2010 = 3.89, SD = 1.69; M2014 = 4.23, SD = 1.56; *p* < 0.001)	Women perceived meat reduction as more beneficial for the environment than men (M = 3.96, SD = 1.69; M = 3.52, SD = 1.70; *p* < 0.001) Larger improvement for women and higher educated participants; *p* < 0.001
Truelove et al., 2012 [26]	Mixed methods. Online survey with open ended questions and behavior ratings.; 2008	USA; Undergraduate psychology majors (*N* = 112) (69 women and 43 men)	Students perceptions of the relative impact and effectiveness of certain behaviors on global warming.	No prior info given.	(1) Open-ended request to participants to list their own behaviors that cause global warming. (2) Respondents asked to rate the impact of 16 behaviors in contributing to GW. Rate went from 1 (Negligible impact) to 11 (Major impact). (3) Open-ended request to participants to list behaviors that reduce global warming. (4) Respondents asked to rate the impact of 20 behaviors that contribute to reduce GW. 1 (Extremely ineffective) to 11 (Extremely effective)	(1) Driving was mentioned by 90% participants. Eat meat only by less than 10% (2) Eat meat was rated with median of 3.83/11, just above behaviors like riding your bike and skiing. SD: 2.52 (3) Drive less and use alternate transportation was mentioned by almost 80% of the participants. Recycle by more than 45%. Reduce meat consumption by less than 5% (4) Reduce your meat consumption: 4.35/11 effectiveness. SD: 2.96	In answer (4), women scored higher than men.
Vanhonacker et al., 2013 [23] *	Online survey; 2011	Belgium (Flanders); convenience sample, *N* = 221, 36% men	Attitudes towards more sustainable food choices and consumer segmentation based on their self-evaluated ecological footprint	Explanation of the concept ‘ecological footprint’ Participants were informed about the contribution of animal production to Co2 emissions.	Participants had to score the contribution to greenhouse gas emissions for various industry sectors, including livestock production. Participants were asked how aware they were of the extent of this contribution.	Approx. M = 3.7 (no number, only bar chart presented) (1 = does not contribute at all to 5 = contributes very much) Livestock production was underestimated relative to other activities (e.g., transport, energy use) 58% reported awareness	n.a.

Notes n.a.: not assessed; M = arithmetic mean; SD = standard deviation; SES = socioeconomic status. *: As reported by [55].

## Appendix B

**Table A2:** People willingness to stop or reduce meat consumption because of its environmental impact.

					Outcome Measure: Willingness to Reduce/Replace		
Author(s), Year	Design; Year Data Collected	Country; Sample	Main Research Question	Provided Information Prior the Experiment	Question or Dependent Variable	Response or Finding	Effect of Covariates
Campbell- Arvai et al., 2014 [29] *	Experimental between- subject design with control group; unspecified.	U.S.; convenience sample of students, *N* = 319, 46% men	Nudging intervention; food- related environmental beliefs and behaviors	Use of a default vegetarian meal option vs. provision of information on the menus.	Hypothetical choice of a lunch or dinner meal (with or without meat)	Offering a vegetarian option as default increased the probability that participants would choose a meat-free meal (OR = 4.10, *p* < 0.001), information on the menu did not significantly influence meal choice (OR = 1.09, *p* = 534).	Females were more likely to choose meat-free menus (OR = 0.49, *p* = 0.02), biospheric value orientation and pro-environmental beliefs were not significant
Cordts et al., 2014 [25] *	Online experiment; 2013	Germany; quota sample, *N* = 590, 52% men	Consumer response to negative information on meat consumption	Randomization to info about negative consequences of meat consumption for animal welfare/health/climate change/personal image; no control group.	Consumers’ belief that they will reduce their meat consumption in the future (measured before and after info)	Before info: 12.8% After info: 18.8% (climate change) to 28.0% (animal welfare)	Condition climate change: Smaller effect in men compared with women (15.5% vs. 22.8%)
De Boer et al., 2018 [38]	Survey data obtained from EU Report; 2012	See EU Report	See EU Report	See EU Report	See EU Report	See EU Report.	(1) Willingness to replace meat (%yes) (2) Willingness to eat less but better meat (%yes) High-income zones Northern zone (1) 38% (2) 77% Western Central zone (1) 42% (2) 78% Medit. zone (1) 55% (2) 86% Medium-to-low income zones Northern zone (1) 54% (2) 83% Western Central zone (1) 63% (2) 86% Medit. zone (1) 57% (2) 86%
Study 1: de Boer, Schösler, et al., 2013 [20] *	Online survey; 2010	Netherlands; quota sample, *N* = 1083, 50% men	Motivational explanations for responses to the meat-free meal idea	As an individual, you can make a big difference to nature and climate protection by choosing one (or more) meals without meat every week.	Willingness to choose one or more meals without meat every week	5% certainly 41% maybe 21% doing so already 23% do not want to	Predictors for ‘does not want to change’ vs. ‘maybe’ (reference): skepticism about climate change (OR = 1.98, *p* < 0.001), value of care for nature (OR = 0.64, *p* < 0.001), level of education (OR = 0.90, *p* < 0.05) (based on standardized predictors)
Study 2: de Boer et al., 2014 [31] *	[the same]	[the same]	Consumers’ strategies to reduce meat consumption and its’ association with their willingness to eat meat-less meals.	[the same]	Willingness to choose one or more meals without meat every week	Same results as in study 1	Predictors for ‘certainly’ vs. ‘maybe’ (reference): Female gender (OR = 2.02, *p* < 0.01), familiarity with topic (OR = 2.67, *p* < 0.001), buying meat substitutes (OR = 1.39, *p* < 0.001), preference for plant-based proteins (OR = 1.34, *p* < 0.01) and number of meat-eating days (OR = 0.70, *p* < 0.001); education and age were n.s.
De Boer et al., 2016 [30]	Nation-wide consumer surveys; 2014	Netherlands and the USA; representative sample *N* = 527 (Netherlands) Weighted variables: gender, age, level of education, region, and a value-related test score on “mentality-environment”. (efficiency of the weighting 89%, effective sample size 478) *N* = 556 (USA). Weighted variables: gender, age, and level of education (efficiency of the weighting 90%, effective sample size 500) Total = 1083	Consumers awareness of meat consumption environmental impact and their willingness to reduce meat consumption, among other research questions.	No prior info given.	Willingness to personally make lifestyle-changes (those already doing it at the time of experiment were instructed to choose the option “certainly willing”).” The answer categories were “Certainly not willing” (1), “Likely not willing” (2), “Likely willing” (4), “Certainly willing” (5), and “Don’t know” (recoded to 3).	Only a small group of participants of both countries were willing to change. Reducing meat consumption was the second less chosen behavior to curb climate change among the DUTCH (M = 3.58 SD = 0.36) and the least chosen among the U.S. (M = 3.01 SD = 1.44)	When participants believed eating less meat to be a highly effective behavior to curb climate change, the medians increased: Dutch M = 4.26 SD = 0.96; U.S. M = 3.88 SD = 1.19.
De Groeve, et al., 2017 [21]	Online survey. Two samples. Data collected in 2015 (sample 1) and 2016 (sample 2)	Belgium; Ghent University Business Administration Students *N* = 429	Assess students support for six less meat initiatives (LMIs) to be implemented in student restaurants.	Each respondent had a 50% chance of receiving information about the climate impact of meat before assessing their support for the LMIs	Support for indirect and direct meat curtailment actions: DIRECT MEAT CURTAILMENT “Eating beef or mutton once a week at maximum.” M “Reduce your portions of meat per meal (for example, 100 g instead of 120 g) P “Increase the supply of vegetarian main meals up to 50% of the meals.” V “Switching to a ‘contrarian week’ in student restaurants whereby meals with meat are served one day a week, and vegetarian meals four days a week.” C	DIRECT MEAT CURTAILMENT STRATEGIES: M Strongly disagree 20% Tend to disagree 27% Neutral 21% Tend to agree 25% Strongly agree 9% P Strongly disagree 9% Tend to disagree 15% Neutral 17% Tend to agree 41% Strongly agree 17% V Strongly disagree 12% Tend to disagree 26% Neutral 28% Tend to agree 24% Strongly agree 10% C Strongly disagree 35% Tend to disagree 33% Neutral 17% Tend to agree 11% Strongly agree 4%	A higher concern for environmental problems is correlated with more positive appraisals of all the LMIs (each *p* < 0.001). A higher KNIM [knowledge about the negative impact of meat **] is also significantly (but less strongly) associated with more positive appraisals of all LMIs, except for LMI-M. Higher appraisals of the direct strategies for meat curtailment (LMIs M, P, V and C) are highly significantly associated with sex and meat consumption frequencies: female students and students who eat meat (or fish) with their main meals less often are more willing to support these LMIs (in every case *p* < 0.001). Prior information about the climate impact of meat appears to have no effect on the support for the LMIs, except for LMI-C, where there is a significant negative effect of information (U = 20,197; *p* = 0.024) **: KNIM’s four themes: environment, animal welfare, health, Global food distribution.
Graca, Calheiros, et al., 2015 [37] *	Study 1: Online survey; 2014	Portugal; convenience sample, *N* = 1023, 42% men	Development and validation of a meat attachment questionnaire	In recent times, meat consumption is being increasingly debated on the grounds of environmental sustainability, health and safety concerns, and animal rights/welfare arguments.	Willingness to reduce meat consumption (1 = not willing at all to 5 = very willing). Willingness to follow a plant-based diet (1 = not willing at all to 5 = very willing)	No mean values presented.	Predictors for meat reduction: Meat attachment (β = −0.49, *p* < 0.001), positive attitudes towards meat (β = −0.11, *p* < 0.05) Predictors for plant-based diet: Meat attachment (β = −0.54, *p* < 0.001), positive attitudes towards meat (β = −0.12, *p* < 0.05), meat consumption frequency (β = −0.12, *p* < 0.01)
Graca, Calheiros, et al., 2015 *	Study 2: Online survey; 2015	Portugal; Amazon Mechanical Turk, *N* = 318, 58% men	Predictive ability of the meat attachment questionnaire for willingness to reduce meat consumption.	see Study 1	Willingness and intention to reduce meat consumption, avoid eating meat, follow a plan-based diet (items averaged for general measure).	No mean values presented	Predictors for willingness: Meat attachment (β = −0.75, *p* < 0.001), PBC (β = −0.12, *p* < 0.01) Predictors for intentions: Attitudes towards meat (β = −0.32, *p* < 0.001), PBC (β = 0.10, *p* < 0.01), meat attachment (β = −0.53, *p* < 0.001).
Graca, Oliveira, et al., 2015 [24]*	Online survey; 2013	Portugal; convenience sample, *N* = 410, 30% men	Multiple correspondence analysis to identify clusters of meat-related associations	Info was provided related to the negative consequences of meat production and consumption for animals, nature and the environment as well as public health	Intent to change current level of meat consumption Willingness to reduce meat consumption by half Willingness to follow a plant-based diet	60% yes, 27% no, (12% no meat consumers) 49% yes, 38% no, (12% no meat consumers) 44% yes, 53% no	n.a.
Hunter et al., 2016 [34]	Postal survey. Date not specified.	Sweden; stratified simple random sample of single family homes. 55% males. 89.5% of the sample had at least one child. Mean age 55. 219 usable questionnaires were returned by post for a response rate of 22% (95% CI (6.25)).	Understand the factors related to fear or danger that motivate consumers to reduce or alter their meat consumption.	Yes, a cover story stating the negative impact of climate change on the earth and humans and statements about the big impact food has on greenhouse gas emissions as well as statement that reducing meat consumption is the most effective food behavior that can be adopted.	Self-efficacy and response efficacy questions regarding meat curtailment strategies	At the same time, the mean scores for self-efficacy and response efficacy show that the participants in this study on average do not find altered meat consumption to be easy, nor do they believe it to be very effective.	
Pohjolainen et al., 2016 [22]	Postal survey; 2010	Finland; representative sample. *N* = 1890	The level of environmental consciousness among Finnish consumers concerning meat production and consumption	No prior info given.	Support to several actions to curb the meat production impact on the environment	Eating less meat the second less supported, only after techno-optimism; only 25.5% considered meat reduction a possible solution. 39.2% rejected this choice.	Consumers clustered in six groups depending on their awareness of meat-related environmental questions: Those aware (highly conscious and rather conscious), those resistant to the idea (Resistant), those who give neutral answers (highly unsure and rather unsure) and those “careless conscious”. Among the highly conscious, 77.2% agree with meat reduction; among the rather conscious, 53% agree with meat reduction.
Schösler et al., 2015 [36] *	Face-to-face interview; 2013	Netherlands; quota samples of second- generation migrants: Turkish/Kurdish *N* = 350, Chinese/Hong Kongese *N* = 350, Native Dutch *N* = 357; 47–49% men	Gender differences in meat consumption and reduction across ethnic group	As an individual, you can make a big difference to nature and climate protection by choosing one (or more) meals without meat every week.	Willingness to reduce meat consumption (including ‘yes’, ‘maybe’)	Willingness to reduce: 17% Turks (monoculture), 53% Chinese (monoculture), 40% Native Dutch	Turkish men followed by Turkish women reported lowest willingness to reduce meat consumption; no gender differences for Native Dutch and Chinese.
Tobler et al., 2011 [33] *	Postal survey; 2010	Switzerland (German- and French-speaking regions); random panel sample, 40% Native Dutch *N* = 6189, 48% men	Consumers’ willingness to adopt ecological food consumption	No info.	Intention assessment based on TTM for eating less meat (maximum once or twice per week)	The largest fraction of unwilling consumers was in the domain of reducing meat consumption. 36.3% (not willing) 5.4% (willing but not ready) 11.4 (willing and ready) 46% (doing it already). Those in the change stages (willing...) were influenced by environmental reasons. Those doing it already were influenced by health reasons.	Female gender (OR = 1.76), importance of naturalness (OR = 1.32), less meat is healthier (OR = 1.21) and better for the environment (OR = 0.87) predicted action state for willingness to reduce meat consumption, all *p* < 0.001; age and education were n.s.
Truelove et al., 2012 [26]	Online survey with open ended questions and behavior ratings.; 2008	USA; Undergraduate psychology majors (*N* = 112) (69 women and 43 men)	Students perceptions of the relative impact and effectiveness of certain behaviors on global warming.	No prior info given.	Respondents asked to rate their intention to perform 20 different proenvironmental behaviors. 1 (Strongly unlikely) to 7 (Strongly likely)	Reduce your meat consumption: 2.99/7 SD:2.07	Effectiveness knowledge did not significantly correlate with intention to perform behaviors that mitigate GW. Effectiveness belief did significantly correlate with the intention to reduce meat consumption.
Vanhonacker et al., 2013 [23] *	Online survey; 2011	Belgium (Flanders); convenience sample, *N* = 221, 36% men	Attitudes towards more sustainable food choices and consumer segmentation based on their self-evaluated ecological footprint.	Explanation of the concept ‘ecological footprint’	Willingness to reduce meat consumption (1 = strongly disagree to 5 = strongly agree)	Meat reduction was rated the most appealing option (approx. M = 3.9, only bar chart shown) out of various options to improve sustainability of food choices (e.g., insects, meat substitutes)	n.a.
EU Report [35]	Telephone survey. 2012	27 EUnion countries; aged 15 and above. In each household, the respondent was drawn at random following the “last birthday rule”. 1000 people sample per country. Small countries: 500 people sample.	EU citizens’ knowledge of green products and their reasons for buying, or not buying, environmentally-friendly products	The interviewer read out: “Some people say large scale meat production has a negative impact on the environment”	Would you be willing to do the following for environmental reasons? (a) Eat less meat but of certified origin (b) Replace most of the meat you eat by vegetables	(a) 80% EU citizens willing to eat less meat but of certified origin Highest: Portugal (89%) Lowest: Estonia (40%) (b) 50% EU citizens willing to replace most of the meat they eat with vegetables Highest: Romania (69%) Lowest: The Netherlands (29%) (Information by country can be found in the report)	The strongest socio-demographic factor linked to willingness to change one’s meat consumption is gender. Female respondents are considerably more willing than male respondents to replace most of the meat they eat with vegetables (59% and 40%, respectively). Women are also more willing to replace beef or pork with poultry or fish (76% versus 67%) and eat less meat but of certified origin (83% versus 76%).

Notes n.a.: not assessed; M = arithmetic mean; SD = standard deviation; SES = socioeconomic status. *: As reported by [55].

## Appendix C

**Table A3:** Vegans, vegetarians, and meat consumption curtailers for environmental reasons.

					Outcome Measure: Reason to Reduce Meat or Become Vegetarian		
Author(s), Year	Design; Year Data Collected	Country; Sample	Main Research Question	Provided Information Prior the Experiment	Question or Dependent Variable	Response or Finding	Effect of Covariates
De Backer, Charlotte J.S. Hudders, Liselot; 2014 [49]	Large-scale Online survey; year not specified.	Belgium; *N* = 1566 (76% women) M age = 26.12 SD = 8.92 10.6% = vegetarians; 41.8% semi-vegetarians; 47.6% light-semi-vegetarians.	Motives underlying the different forms of vegetarianism and semi-vegetarianism in a culture where meat continues to play a crucial role in people’s diets.	No prior info provided.	Agree or disagree with a 7-point Likert scale with motives for meat reduction/avoidance. Ecological motives: “I don’t eat meat every day because it is better for the environment,” and “I don’t eat meat every day because eating meat increases my ecological footprint”.	143/165 vegetarians strongly agreed with ecological motives (6.1 or higher in a Likert scale 1–7). For 28/143 ecological concerns were the main drive (mean of 6.5/7 Liker scale) The rest of the vegetarians (*n* = 22) disagreed with the ecological concerns (mean of 2.61/7 Likert scale). 323/650 semi-vegetarians: reported ecological concerns as the main motivator for strongly reducing meat. (Mean of 5.57/7 Likert scale) 254/741 light semi-vegetarians reported ecological concerns as the main motivator for avoiding meat one or two days a week. (Mean of 5.12/7 Likert scale)	Ecological concern positively associated with meat reduction, except for light semi-vegetarians.
De Boer et al., 2017 [52]	Face-to-face interviews; 2013	Netherlands; two samples of adults (aged 18–35) Native Dutch, *n* = 357, (Men 48%) Second generation Chinese Dutch, *n* = 350 (Men 47%) Participants were categorized in four dietary groups (all self-declared) (1) Vegetarians (2) Low meat eaters (2–3 days a week) (3) Medium meat eaters (4–5 days a week) (4) High meat eaters (6 days or more)	Differences between vegetarians and three categories of meat eaters in relation to (1) key characteristics of their hot meal, (2) strength and profile of their food-related motivation, and (3) reasons for and reasons against frequently eating meat?	No prior info provided.	Indicate three reasons for not frequently eating meat. Among them, participants could choose “Because it’s better for the environment”.	NATIVE DUTCH; Self-declared vegetarians: 21% indicated the environment as a reason for not frequently eating meat. Low meat-eaters: 30% Medium meat-eaters: 44% High meat-eaters: 41% TOTAL: 38% CHINESE DUTCH: Self-declared vegetarians: 42% Low meat-eaters: 38% Medium meat-eaters: 32% High meat-eaters: 15% TOTAL: 26% Environmental and financial reasons were mentioned relatively often, but according to the authors, the fact that they were also mentioned by high meat-eaters indicates that, under the current circumstances, these reasons are not decisive for a reduction in meat consumption.	Native Dutch: the more meat they eat, the more they would give an environmental reason for not eating meat. Chinese Dutch, the less meat they eat, the more report the environment as reason for not eating meat. In both samples, the vegetarians were more often women (about 70%), whereas the high meat-eaters were more often men (about 70%).
Dyett, Patricia A., et al., 2013 [40]	Postal survey; (year not reported)	United States; *N* = 100 Population of self-reported vegans for more than 9 months living in different U.S. States. Age: 25–75 yrs old Vegans defined as individuals who used no meat, fish, or poultry, and who used dairy- or egg-containing products less than once per month.	Discover the main reasons for adopting and maintaining a vegan lifestyle and to determine whether participants’ diet and lifestyle choices coincided with positive health indices and selected outcome assessment.	No prior info provided.	Reason for being vegan	Because environmental values (2%)	n.a.
Turner-McGrievy, G. et al., 2016 [41]	online quota survey; year not specified;	Majority (90%) from the United States; *N* = 422 (*n* = 125 ULTRA, *n* = 152 FULL, *n* = 145 HALF) More ULTRA participants were men (63%) (vs. FULL (37%) and HALF (23%)	Examine differences in current vegetarian and vegan diets, reasons for it and other dietary behaviors among long distance runners.	No prior info provided.	Participants asked to select all reasons for choosing their current diet that apply to them from a list of 12 reasons (including an option to select no reason or to write in an answer).	More ULTRA participants (*n* = 25, 20%) reported that environmental concerns shaped their diet choice as compared with FULL and HALF participants (*n* = 36, 12%; χ2 = 4.4, *p* = 0.04).	n.a.
Haverstock, Katie, et al., 2012 [48]	Food Choice Questionnaire; year not specified;	International online sample; *N* = 247 (196 = current animal product limiters and 51 former limiters) 211 = females; Age = 18 to 66 (M = 29.05, SD = 9.39) 222 = Euro-Americans.	Similarities and differences between current and former animal product limiters.	No prior info provided.	Eight items concerning ethical food choice motives were also included [...] These ethical motives include animal welfare, environmental protection, political values, and religion. Likert scale: 1 = not important to 4 = very important.	Importance given to environmental reasons to reduce or avoid meat. CURRENT LIMITERS: Vegans (*n* = 119) M = 3.10, SD = 0.68 Vegetarian (*n* = 54) M = 2.71, SD = 0.74 Pescatarian (*n* = 22) M = 2.79, SD = 0.75 FORMER LIMITERS: Now a regular meat eater (*n* = 16) M = 2.13, SD = 0.94 Now a occasional meat eater (*n* = 26) M = 2.67, SD = 0.80 Now a meat avoider (*n* = 4) M = 2.17, SD = 0.43 Now a pescatarian (*n* = 5) M = 2.54, SD = 0.88	Few gender differences. Women more strongly endorsed health and the environment motives than did men.
Hoffman, Sarah R. et al., 2013 [39]	Online survey; 2011	USA; People recruited through Facebook, Google, and vegetarian dedicated webpages. *N* = 312 Age: 18–69. (42% = age 20–29) 15.4% men, 84.6% women. 68.3% had some form of Higher Education. 86.5% White-Caucasian 56.7% had an income of <49,000 USD Vegetarian 49.4 (vegetarian) and 50.6 (vegan).	Examine the differences between health and ethical vegetarians by comparing conviction, nutrition knowledge, dietary restriction, and years as vegetarian between the two groups.	No prior info provided.	In order to place subjects into categories (i.e., health, ethical, or other), two multiple choice items were created: “The main reason I became a vegetarian was because of (check only one),” “The main reason I am (still) a vegetarian is because of (check only one).” Fourteen options were given in addition to the option “other”	234 = ethical reasons (animal, ethics, religion, environment) (10 = the environment) as initial reason to become vegetarian.	Not reported.
Izmirli, et al., 2011 [51]	Survey; year not specified;	11 Eurasian countries; *N* = 3433 university students from 103 universities. 47% avoided some meat products. 4% vegetarians 0.4% vegans	Determine the relationship between the consumption of animal products and attitudes towards animals among university students in Eurasia	No prior info provided.	Specify the major reason for meat avoidance like health concerns, religious instruction, concerns for the suffering of animals or for the environment.	479 students (38.1%) gave the environmental reason.	Among “some meat avoidants” (total = 1147) 468 41% because of the environment. (Most chosen reason). Among “vegetarians” (total = 99) 9 (9%) because of the environment. Among “vegans” (total = 7) 2 (29%) because of the environment.
Lindeman, Marjaana, et al., 2001 [44]	STUDY 1 Food Choice Questionnaire; year not specified.	Finland; 82 female participants. Age: 17–3 years old. 30.4% semi-vegetarians and 25.3% vegetarians.	The construction of food choice ideologies and the ways dietary groups endorse them.	No prior info provided.	Food Choice Questionnaire. Motives assessed among others: ecological welfare (including animal welfare and protection of nature). Subjects had to rate the statement “It is important to me that the food I eat on a typical day...” on a 4-point scale (1 = not at all important, 4 = very important).	Ecological welfare. Semi and full vegetarians: M = 3, SD = 0.74	Vegetarians regarded ecological food choice reasons as more important than semivegetarians did, *t*(45) = −4.12, *p* < 0.001.
Lindeman, Marjaana, et al., 2001	STUDY 2 Food Choice Questionnaire; year not specified.	Finland; *N* = 149 women. Age: 19–74 Mean age: 31.5. 44.3 full time students. 41.6% employed women. 16.8% semivegetarians and 10.7% vegetarians.	Idem	No prior info provided.	Idem	Ecological welfare. Semi and full vegetarians: M = 2.94, SD = 0.80	n.a.
Péneu, et al., 2017 [50]	Online survey. Ongoing web-based prospective observational cohort study launched in France in May 2009 with a scheduled follow-up of 10 years.	France; *N* = 22,935 (5688 men)	Investigate the sociodemographic profiles of individuals reporting health and environmental dilemmas when purchasing meat, fish and dairy products, and compare diet quality of individuals with and without dilemma.	No prior info provided.	Respondents have to agree or disagree with the following statement: “I avoid purchasing [meat/fish/dairy products] for environmental issues”	25% strongly agree or agree	
Péneu, et al., 2017					Asked to agree or not with “I am torn between purchasing [meat/fish/dairy products] to follow dietary guidelines or limit purchase for environmental issues”.	31.94% said YES	- Women declared more dilemma in the case of meat than men. - In the case of meat, individuals with greater educational level and household including only one adult were more likely to report a dilemma.
Povey et al., 2001 [53]	Open ended questionnaires; year not reported.	United Kingdom; Convenience sample; 111 respondents (25 meat eaters, 26 meat avoiders, 34 vegetarians, 26 vegans).	Examine differences between the attitudes and beliefs of four dietary groups (meat eaters, meat avoiders, vegetarians and vegans) and the extent to which attitudes influenced intentions to follow a diet.	No prior info provided.	Record salient thoughts, beliefs and feelings towards these three diets: meat, vegetarian and vegan. A maximum of eight thoughts, beliefs or feelings could be recorded by participants.	MEAT DIET: 6/26 vegans and 6/26 meat avoiders named environmental problems as a salient belief towards eating a meat diet. VEGETARIAN DIET: 4/26 vegans mentioned a vegetarian diet to be environmentally friendly. VEGAN DIET: 12/26 vegans mentioned it to be environmentally friendly.	n.a.
Pribis, et al., 2010 [43]	cross-sectional, observational study; 2007	United States; Andrews University (SDA institution) undergraduate students and their respective families. *N* = 609 participants. (35% male) Mean age = 31 years old. 4% vegans; 25% lacto-ovo vegetarians; 4% pesco-vegetarians; 67% non-vegetarians.	Examine whether reasons to adopt vegetarian lifestyle differ significantly among generations.	No prior info provided.	Using a Likert Scale from 1 to 5 (strongly disagree [1]–agree [2]–no opinion [3]–agree [4]–strongly agree [5]) participants rated reasons why they choose a vegetarian lifestyle. Vegetarian reason: “vegetarian lifestyle is much more protective against the environment”	Responses across generations: 11–20 years old: 3.95 21–40 years old: 3.69 41–60 years old: 3.75 61 or older: 3.79	Younger people (11–20 years) also significantly agreed more with the environmental reason (*p* = 0.025).
Rozin, et al., 1997 [45]	Questionnaire; 1987	United States; *N* = 104 self-identified as at least reluctant to meat. 34 = male Mean age: 26.6 (SD = 8.95)	Describe moralization in the domain of vegetarianism.	No prior info provided.	A list of 20 possible reasons for avoiding meat. Subjects indicated both current agreement (5-point scale ranging from disagree strongly to agree strongly) with each reason and, if relevant, the time of onset of the reason (“this was your first reason for avoiding meat,” “this was one of the earliest reasons for avoiding meat,” “this was not one of the earliest reasons for avoiding meat,” or “this was never a reason for avoiding meat”). Ecological reason: “I resist [avoid] eating “meat” because it is wasteful of resources to eat animal rather than vegetable products, especially in a world where people are starving.”	5.8% “initial reason” to avoid meat. 38.2% strongly agreed (22.5% agreed) with ecological reason as current reason.	n.a.
Schösler et al., 2015 [36] *	Face-to-face interview; 2013	Netherlands; quota samples of second- generation migrants: Turkish/Kurdish *N* = 350, Chinese/Hong Kongese *N* = 350, Native Dutch *N* = 357; 47–49% men	Gender differences in meat consumption and reduction across ethnic group		Reasons for not frequently eating meat (selection of maximum 3 reasons out of 9 reasons)	It’s better for the environment’ was selected by 2% Turks, 26% Chinese, 38% Native Dutch	
Study 3: Schösler, de Boer, & Boersema, 2014 [46] *	Online survey; 2010	Netherlands; quota sample, *N* = 1083, 50% men	Cluster analysis based on type of eating-related motivation and profiling of segments related to meat consumption	No info before questioning	Reasons for not frequently eating meat (selection of maximum 3 reasons out of 9 reasons)	19% selected ‘It’s better for the environment’	34% of those consumers who internalized the importance of the food-nature relationship agreed that eating less meat is better for the environment.
Vanhonacker et al., 2013 [23] *	Online survey; 2011	Belgium (Flanders); convenience sample, *N* = 221, 36% men	Attitudes towards more sustainable food choices and consumer segmentation based on their self-evaluated ecological footprint.	Explanation of the concept ‘ecological footprint’	Participants had to indicate environmentally-friendly behaviors (what they actually do)	4% consume less meat per meal to reduce their ecological footprint	n.a.
Verain et al., 2015 [47] *	Online survey; 2011	Netherlands; quota sample, *N* = 942, 50% men.	Segmentation of consumers based on sustainable food behaviors and profiling of segments	No info	Performance of sustainable food behaviors at least once a month in the previous year (‘yes’, ‘no’).	One meat-free day a week (56%) and smaller meat portions (52%) were the most popular sustainable food behaviors compared with other behaviors (e.g., buying organic meat or dairy)	Female gender (β = 0.08, *p* < 0.001), age (β = 0.21, *p* < 0.05) and variables on personal/social norms and subjective knowledge about sustainable food choices positively predicted curtailment behavior (average of four items: eating smaller portions of meat, eat less diary, eating less, one meat-free day a week)
White et al. 1999 [42]	Survey; date not specified	United States; Random sample. *N* = 2500 women from each of the past decades’ graduating medical school classes 8% Self-described vegetarians	Investigate the prevalence and characteristics of vegetarian subjects in the Women Physician’s Health Study and compare them with the omnivore cohort.	No prior info provided.	Self-categorized vegetarians were asked why they were vegetarian.	32.1% cited environmental reasons.	n.a.

Notes n.a.: not assessed; M = arithmetic mean; SD = standard deviation; SES = socioeconomic status. *: As reported by [55].
